# Bacteria Isolated from Bats Inhibit the Growth of *Pseudogymnoascus destructans*, the Causative Agent of White-Nose Syndrome

**DOI:** 10.1371/journal.pone.0121329

**Published:** 2015-04-08

**Authors:** Joseph R. Hoyt, Tina L. Cheng, Kate E. Langwig, Mallory M. Hee, Winifred F. Frick, A. Marm Kilpatrick

**Affiliations:** Department of Ecology and Evolutionary Biology, University of California Santa Cruz, Santa Cruz, California, United States of America; University of Regina, CANADA

## Abstract

Emerging infectious diseases are a key threat to wildlife. Several fungal skin pathogens have recently emerged and caused widespread mortality in several vertebrate groups, including amphibians, bats, rattlesnakes and humans. White-nose syndrome, caused by the fungal skin pathogen *Pseudogymnoascus destructans*, threatens several hibernating bat species with extinction and there are few effective treatment strategies. The skin microbiome is increasingly understood to play a large role in determining disease outcome. We isolated bacteria from the skin of four bat species, and co-cultured these isolates with *P*. *destructans* to identify bacteria that might inhibit or kill *P*. *destructans*. We then conducted two reciprocal challenge experiments *in vitro* with six bacterial isolates (all in the genus *Pseudomonas*) to quantify the effect of these bacteria on the growth of *P*. *destructans*. All six *Pseudomonas* isolates significantly inhibited growth of *P*. *destructans* compared to non-inhibitory control bacteria, and two isolates performed significantly better than others in suppressing *P*. *destructans* growth for at least 35 days. In both challenge experiments, the extent of suppression of *P*. *destructans* growth was dependent on the initial concentration of *P*. *destructans* and the initial concentration of the bacterial isolate. These results show that bacteria found naturally occurring on bats can inhibit the growth of *P*. *destructans in vitro* and should be studied further as a possible probiotic to protect bats from white-nose syndrome. In addition, the presence of these bacteria may influence disease outcomes among individuals, populations, and species.

## Introduction

Emerging infectious diseases can have devastating impacts on wildlife, and they currently threaten many species with extinction [1–4]. With an increase in anthropogenic disturbance and rise in global trade and transportation, the threat posed by wildlife disease is likely to increase [5]. Wildlife diseases can be exceedingly challenging to manage because free ranging animals are difficult to treat with drugs or vaccines, and many strategies require constant human intervention[6]. For example, the re-establishment of Black-footed ferrets into their native range required vaccination of adults and young born each year for both plague and canine distemper [7]. New approaches that do not require continued intervention are needed to reduce the impacts of these devastating diseases [8].

Several recently emerged wildlife pathogens infect host dermal tissue, and interactions with host skin microbiota could play an important role in disease severity. Vertebrate skin is an ecosystem composed of different habitats which harbor diverse assemblages of microorganisms [9]. Previously, studies of skin microbiota primarily examined the pathogenic roles of skin microbes, with little attention to the beneficial function that many microorganisms may provide [10]. However, beneficial bacteria on skin can provide vital functions to their hosts, including processing of skin proteins, freeing fatty acids to reduce invasion of transient microorganisms, and inhibition of pathogenic microorganisms [11]. Some bacteria, termed probiotics, or beneficial bacteria [12], have been developed to reduce the impact of a broad range of diseases.

Probiotics that can establish on hosts have the potential to provide a long-lasting solution for managing disease and, unlike chemical fungicides, may be able to coevolve with a pathogen [13]. Probiotics are regularly used in the biological control of disease in both aquaculture and agriculture, but have yet to be widely implemented in controlling wildlife disease, possibly because of perceived risks and lack of demonstrated success [14–17]. Risks associated with augmenting micro-organisms on a host, which can either be ineffective or accidentally harmful, can be minimized by using bacteria that naturally occur in the hosts’ environment [18]. Resistant or tolerant species that are phylogenetically closely related to a heavily impacted species may host bacteria that could serve as probiotics, and these bacteria may be more likely to be able to colonizing the target species’ skin [18].

Here, we assess whether bacteria naturally occurring on bats can reduce the growth of *Pseudogymnoascus destructans*, the pathogen that causes white-nose syndrome (WNS)[19]. White-nose syndrome first emerged in Howe’s Cave, New York, in 2006, and spread quickly, causing precipitous declines in hibernating bats throughout Eastern North America[2,20]. Four species (*Myotis septentrionalis*, *Myotis sodalis*, *Myotis lucifugus*, and *Perimyotis subflavus*) have suffered >90% declines in regional populations and one species, *M*. *septentrionalis*, is on a trajectory towards extinction [2,20]. *Myotis septentrionalis* has recently been proposed by the U.S. Fish and Wildlife Service for listing under the Endangered Species Act and has been listed under the Canadian Species at Risk Act as Endangered.

*Pseudogymnoascus destructans* infects the dermal tissue of bats and grows optimally between 10–14°C [21], similar to the temperature of hibernating bats. *Pseudogymnoascus destructans* infection may disrupt bats’ physiological processes including heat and water loss and electrolyte balance [22], and typically results in increased arousal frequency by hibernating bats [19,23]. Increased arousal frequency may prematurely deplete bats’ fat stores resulting in death approximately 70–120 days after infection, based on laboratory infection trials[19,22,24]. Individuals able to survive through hibernation until spring appear to clear infection and fully recover [24,25]. However, these bats become re-infected the following fall, in part because *Pseudogymnoascus destructans* is capable of persisting for long periods of time in the absence of bats [26,27].

Currently, there are few management options that can reduce mortality in affected regions [28]. Preliminary investigations of treatments to reduce mortality using antifungal drugs caused higher mortality in the treated groups than control groups, possibly due to toxicity [29]. Other proposed chemical treatment options have avoided the toxic effects of the direct application of chemicals, but have yet to be validated *in situ* [30]. Thus, treatment options are urgently needed, and a probiotic may be an effective way to reduce WNS impacts if it could at least partially inhibit *P*. *destructans* growth and delay mortality long enough for bats to survive hibernation.

We cultured bacteria isolated from the skin of four species of hibernating bats from eastern North America to determine whether naturally occurring bacterial species might exist within the skin microbiome of bats that could inhibit growth of *P*. *destructans*. We then quantified the anti-fungal efficacy of these bacteria across a range of concentrations in two challenge experiments.

## Methods

### Sampling and isolating cutaneous microbes

We conducted sampling for cutaneous bacteria on hibernating bats at two hibernacula in New York and two in Virginia (exact locations of study sites are not provided to protect sensitive wildlife habitat). We rubbed sterile polyester swabs dipped in sterile water back and forth five times along each bat’s forearm and muzzle. Swabs were frozen in 20% glycerol stock for later culturing. We collected swabs from ten individuals from each of four species *Eptesicus fuscus*, *Myotis leibii*, *M*. *lucifugus*, and *M*. *sodalis*.

Epidermal swab sample collection protocols for this study were approved by the University of California, Santa Cruz IACUC (protocol # FrickW1106). Sample collection was permitted by authorized state biologists from the New York Department of Environmental Conservation and Virginia Department of Game and Inland Fisheries. Handling and sampling of endangered species (*Myotis sodalis)* was conducted under the appropriate state and federal permits.

Each swab was plated on two types of general media, Reasoner’s 2A agar (R2A) and sabouraud dextrose agar (SDA), and plates were incubated at 9°C for three weeks. We classified bacteria on each plate by morphotype, using color, growth form, and gram staining techniques. We isolated one colony from each sample by morphotype (to reduce repeat sampling of the same isolate) using a sterile inoculating loop and re-plated each isolate on R2A media and grew them for 2–5 days at 9°C. Each isolate was cryo-banked by sampling from each of these colonies with a sterile inoculating loop, placing the sample in 30% glycerol, and freezing it at -80C for later use.

### Pre-screen for bacteria with anti-*P*. *destructans* properties

We determined whether isolates could inhibit the growth of *P*. *destructans* using a challenge protocol adapted from the National Committee for Clinical Laboratory Standards. All culturing was done on SDA. [31]. A suspension containing 1.7 x 10^7^ *P*. *destructans* conidia/ml (quantified using a hemocytometer) was prepared by flooding a 3 week-old culture of *P*. *destructans* grown on SDA with 20 ml of 1X phosphate buffered saline with tween_20_ (PBST_20_). Colonies were submersed for 5 minutes, and then gently rubbed with a sterile inoculating loop to free the conidia. The supernatant was drawn off and placed into a 50ml falcon tube and vortexed to homogenize the suspension. Each 90mm plate was inoculated with 200ul of the *P*. *destructans* suspension and allowed to air dry for 10 minutes. We added bacteria on the plate already inoculated with *P*. *destructans* from a growing culture using pinpoint inoculation at three equally spaced points on top of the dried *P*. *destructans* suspension. Bacteria cultures were grown from frozen stock 24 hours earlier on SDA. Plates were placed into incubators at 9–10°C, which is within the range that bats hibernate [32], and growth was monitored every other day for 14 days and on day 14 any bacteria that produced a zone of inhibition (a visible reduction of *P*. *destructans* growth surrounding the bacterial colony) were included in subsequent challenge experiments described below In addition to bacterial isolates from bats, a *Pseudomonas fluorescens* isolate *Pf*A506 commonly used in biocontrol of agricultural fungal pathogens was included as a positive control [33], and two types of negative controls, 1) a sham inoculation with 20% sterile glycerol stock, and 2) two bacteria isolated from bats in the genera *Chryseobacterium* and *Sphingomonas* (both gram-negative rod shaped bacteria) that are not known to produce anti-fungal compounds [31].

### Identification of bacterial isolates

We identified bacterial isolates used in the following inhibition assays using PCR amplification and DNA sequencing. DNA for PCR was obtained by suspending a small amount of a bacterial colony in 100 μl of sterile deionized water (SDW) and lysing the cells at 95°C (10 min). Universal bacterial 16S rRNA gene primers (16S_F (5`- ACC GCG ATA ATA CGT CCC GAT CG—3`) and 16S_R(5`- TGC GGA CGT GAA GTG CTA G -3`)) were used to amplify the ~1.5 kb 16S rRNA gene fragment [34]. The following was added to each PCR template: 1 μl of crude lysate DNA template, 1.5 μl of each 0.6 μM forward and reverse primer, and 5 μl of Taq 5X MM (NEB) at 1X concentration, which contains 1.5 mM MgCl2, 2 mM dNTPs, and PCR buffer. Reaction volumes were made up to 25 μl with SDW. The reaction conditions involved an initial denaturation at 95°C for 3 minutes, followed by 35 cycles of denaturation at 95°C for 15 sec, primer annealing for 15 seconds at 49°C, and extension for 90 seconds at 42°C. The 16S rRNA gene sequences were compared with known sequences in the EMBL database using MEGA BLAST (BLASTN 2.1.1, [35]) to identify the most similar sequence alignment. *Pseudomonas fluorescens* isolate *Pf*A506 was used to assure proper alignment of sequences.

### Inhibition Assays

Two separate inhibition assays were performed. In the first inhibition assay, we determined the ability of each bacterial isolate to grow on lawns of different starting concentrations of *P*. *destructans*, and to produce a zone of inhibition in which *P*. *destructans* growth was either delayed, halted adjacent to the bacteria colony. In the second inhibition assay, we measured the growth of *P*. *destructans* on a lawn of different starting concentrations of each bacterial isolate.

In the first inhibition assay, the growth of each bacterial isolate was quantified in media inoculated with four *P*. *destructans* concentrations estimated using hemocytometry (10^7^, 10^6^, 10^5^, and 10^4^ conidia/ml). After the *P*. *destructans* suspension was dry and conidia fixed to the plate, we used a pipette to inoculate the plates with 0.1 μl of a 10^8^ cfu/ml suspension of a given bacterial isolate at three evenly spaced points on the plate. The bacterial suspension was prepared by suspending whole colonies in 30% glycerol, and using an inoculating loop to suspend the colony. Each treatment was replicated nine times, and cultures were grown at 9°C for 37 days. Zones of inhibition were quantified by measuring the distance from the edge of the bacterial colony to the edge of the visible *P*. *destructans* growth every other day [31]. We also microscopically examined the zones of inhibition on the final day of the experiment (day 43) to characterize the effects of bacteria on the growth of *P*. *destructans*.

In the second inhibition assay we determined the ability of each bacterial isolate to prevent growth of *P*. *destructans* across a series of six bacterial concentrations. Each bacterial isolate was plated from cryobanked glycerol stock onto a Petri dish with SDA media and allowed to incubate for two days at 9°C before being added to a 30% sterile glycerol suspension. The concentration of each isolate was standardized by making serial ten-fold dilutions of the culturing stock and then counting the number of colony forming units (cfu) per ml. The bacteria glycerol suspension was frozen at -20°C while calculating the concentration. Each stock was standardized to the same concentration of 7.5x10^7^ cfu/ml using 30% glycerol. We plated 50 μl of each bacterial glycerol dilution on SDA in 60 mm Petri dishes. We used three replicates per treatment for bacterial concentrations 10^6^, 10^2^, and 10^1^ cfu/ml, and five replicates for concentrations 10^5^, 10^4^, and 10^3^ cfu/ml. For the control plate, we added 50 μl of sterile 30% glycerol solution to the plates and then inoculated with *P*. *destructans* using a pinpoint inoculation. The diameter of the *P*. *destructans* colony was measured for a total of 42 days. Measurements were made every other day for the first 14 days, and then once every seven days thereafter until the end of the experiment.

We used cell-free supernatant plated on a lawn of *P*. *destructans* to determine if anti-fungal compounds were being produced by the bacteria in the initial culture. We used cultures of fresh bacteria grown in isolation, and co-cultured with *P*. *destructans*, in lysogeny broth. Cultures were then centrifuged for 30 minutes and the supernatant was drawn off. We then inoculated three plates with 50 μL of the supernatant on a lawn of *P*. *destructans* using the methods described above.

### Bacteria motility and chemotaxis

Bacteria motility experiments were conducted to assess whether the *Pseudomonas* isolates from bats preferentially move towards *P*. *destructans*. A 0.3% agar SDA media was prepared and a sterile inoculating loop was dipped into a 7.5x10^7^ cfu/ml of bacterial suspension and then stabbed ~5 cm into the soft agar for all nine bacterial isolates. To determine whether or not the bacteria preferentially moved towards *P*. *destructans*, we repeated the same methods described above, but included a small colony of *P*. *destructans* that was stabbed into the media on one side of the tube. The tubes were incubated for 1 week at 10°C, and then stabs were visually inspected for signs of whether bacteria moved away from the initial stab or moved towards the *P*. *destructans* stab.

### Statistical analysis

We fit linear mixed effects models (function glmer in package lme4 [36] in R v. 3.02 [37]) with day as a categorical random effect and bacteria type and concentration as fixed effects to examine the influence of bacteria type and serial dilution on the zone of inhibition (first inhibition assay, S2 Table) and diameter of fungal colony (second inhibition assay, S3 Table). We fit five a priori models including additive and interactive effects (S4 Table) and compared models using Akaike’s Information Criterion (AIC) [38].

## Results

### Pre-screen for bacteria with anti-*P*. *destructans* properties

We isolated a total of 133 bacterial morphotypes from the 40 bats we swabbed. Four isolates from *E*. *fuscus* (from 3 bats) and two isolates from two individual *M*. *lucifugus* inhibited *P*. *destructans* growth in standard challenge assays (Table 1). These six bacteria were selected for further testing in the two inhibition assays. All were identified as members of the genus *Pseudomonas*, with five of the six isolates most closely related to the *Pseudomonas fluorescens* group and the other isolate (PA6) being most closely related to *Pseudomonas abietaniphila* (HF952541) (S1 Table).

**Table 1:** Bacteria isolated from *Myotis lucifugus* and *Eptesicus fuscus* used in challenge experiments.

*Graph ID*	*Bat Species*	*Collection County*	*Collection Date*	*Phylogenetic affiliation*	*Motile*
*CHR1*	*M*. *lucifugus*	Ulster, NY	8-Apr-12	*Chryseobacterium sp*.	No
*SPH2*	*E*. *fuscus*	Bath, VA	29-Mar-12	*Sphingomonas sp*.	No
*PF1*	*E*. *fuscus*	Bath, VA	28-Mar-12	*Pseudomonas sp*.	Yes
*PF2*	*E*. *fuscus*	Bath, VA	29-Mar-12	*Pseudomonas sp*.	Yes
*PF3*	*M*. *lucifugus*	Highland, VA	19-Mar-12	*Pseudomonas sp*.	Yes
*PF4*	*M*. *lucifugus*	Bath, VA	29-Mar-12	*Pseudomonas sp*.	Yes
*PF5*	*E*. *fuscus*	Albany, NY	Jan-09	*Pseudomonas sp*.	Yes
*PA6*	*E*. *fuscus*	Bath, VA	29-Mar-12	*P*. *abietaniphila*	Yes
*PF7*	NA	NA	NA	*P*. *fluorescens A506*	Yes

### Inhibition assays

In the first inhibition assay, bacterial colonies initially expanded quickly and then plateaued in size, with growth continuing for longer at lower initial concentrations of *P*. *destructans* in the media (S1 Fig., S4 Table). Some bacterial isolates formed much larger colonies than others, with PF1, PF2, and PF4 forming the largest colonies (S1 Fig.). The size of bacterial colonies increased with decreasing initial concentrations of *P*. *destructans* (S1 Fig., S4 Table).

**Fig 1:**
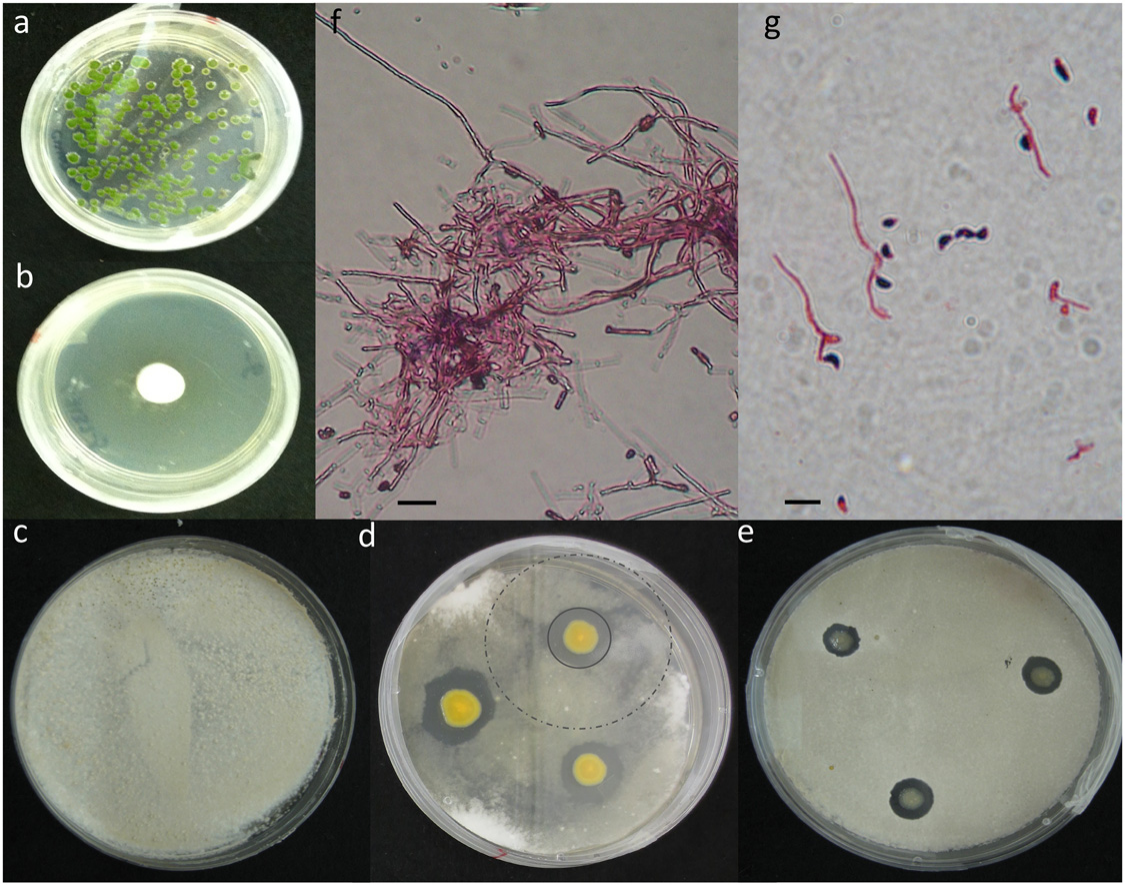
Challenge plates showing the inhibition of *Pseudogymnoascus destructans*. Bacteria were plated with an initial starting concentration of 10^4^ cfu/ml (PF2). The plate (a) shows no visible *P*. *destructans* growth on day 43, compared to the (b) control plate showing uninhibited *P*. *destructans* colony growth at day 43. (d) The zones of inhibition produced by one of the top performing *P*. *fluorescens* isolates (PF2) compared to the sham inoculated control (c) and a widely used strain of *P*. *fluorescens*, (e; PF7: *Pf*A506). There are two distinct zones of inhibition produced by the top performing strain (as shown in panel d) indicated by the grey solid circle and the dashed grey circle. Microscopic images of the inner and outer zones are shown in panels (f) and (g). We used gram staining techniques to help better visualize conidia (purple) and hyphae (pink) (scale bars, 10 μm). Within the first zone, indicated by the dark ring surrounding the yellow bacteria colony (PF2), the bacteria either arrested or delayed conidia growth, (g) which can be seen by the small hyphael extension from the conidia. Outside of this first zone, the growth of *P*. *destructans* was much more extensive (f), producing a mycelial network before its growth was arrested.

**Fig 2:**
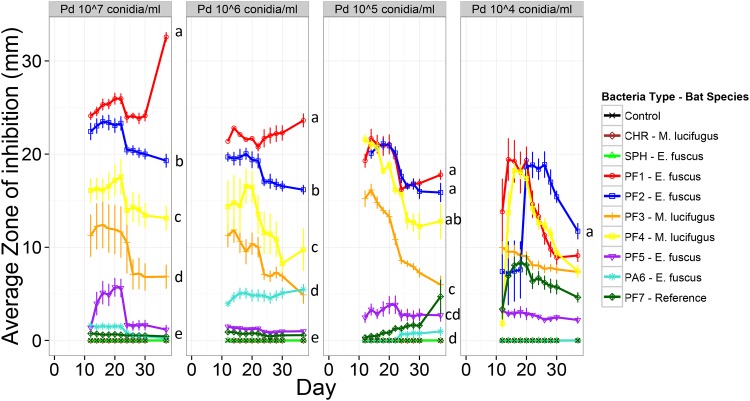
First inhibition assay measuring the width of the zone of inhibition produced by bacteria on a lawn of *P*. *destructans*. The zones of inhibition produced by bacterial isolates when inoculated on a plate with four concentrations of *P*. *destructans*. Lines denoted by the same letter do not differ significantly on the last day of the experiment (S5 Table). CHR and SPH are isolates in the genus *Chryseobacterium* and *Sphingomonas* that are not known to produce antifungal compounds. The control is an inoculation of 30% glycerol stock. PF1-5,7 and PA6 are all isolates in the genus *Pseudomonas*.

Zones of inhibition could not be visualized until *P*. *destructans* growth was visible on days 9–11 (Fig. 1). At this time, zones of inhibition already differed significantly among bacterial isolates and initial concentrations (Fig. 2, S2 Fig., and S5 Table). Two bacterial isolates, PF1 and PF2, generated larger zones of inhibition across most initial concentrations of *P*. *destructans* by the end of the experiment (S2 Table). Three isolates (PF1, PF2, and PF7) established two zones, one where growth of *P*. *destructans* was suspended immediately upon germination (Fig. 1g), and another outside of this zone where growth was arrested, but only after the mycelial mat had begun to develop (Fig. 1f). Zones of inhibition on the last day of the experiment (day 37) increased with increasing initial concentrations of *P*. *destructans* for the *Pseudomonas* isolates showing the strongest inhibition (Fig. 2; PF1, PF2, and PF4; all concentration slopes were significantly negative, all p-values <0.03). For the other four *Pseudomonas* isolates, the zone of inhibition was either variable across concentrations (Fig. 2; PF3, PF5, PA6) or increased with decreasing initial *P*. *destructans* concentration (PF7; concentration coef. 1.72 ± 0.64, p = 0.008). Two isolates, PF1 and PF2, out-performed the reference *P*. *fluorescens* strains (PF7; *PfA506*) at all initial concentrations with at least a two-fold difference in zone of inhibition (Fig. 2). The two control bacteria (*Chryseobacterium sp*. *and Sphingomonas sp*.*)* and the sham-inoculated control produced no zones of inhibition (Fig. 2).

**Fig 3:**
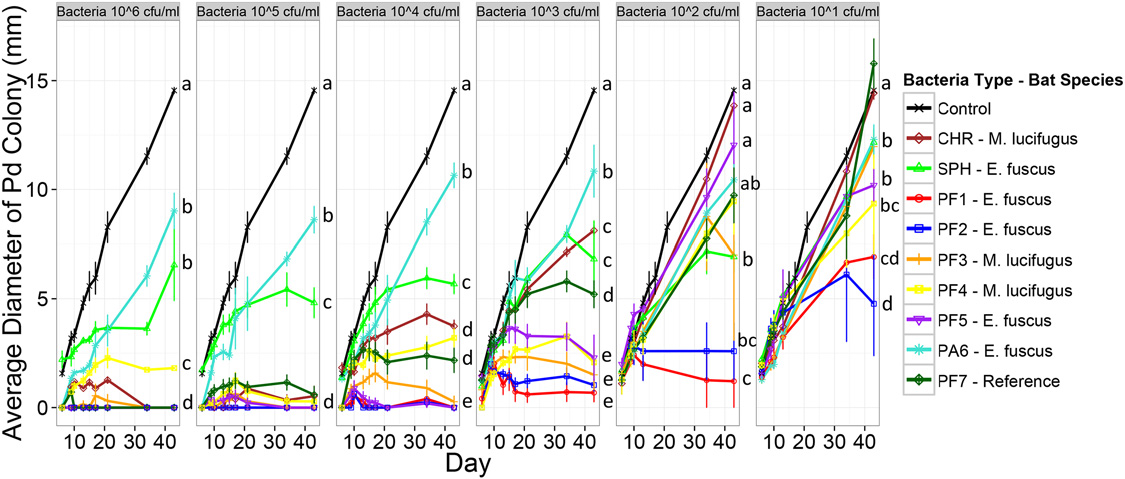
Second inhibition assay measuring the diameter of *P*. *destructans* colonies grown on a lawn of bacteria. *Pseudogymnoascus destructans* was plated with nine bacterial isolates at six different concentrations (highest to lowest, left to right). Lines denoted by the same letter did not differ significantly on the last day of the experiment. CHR and SPH are isolates in the genus *Chryseobacterium* and *Sphingomonas* that are not known to produce antifungal compounds. The Control is a sham inoculation of 30% glycerol stock. PF1-7 and PA6 are all isolates in the genus *Pseudomonas*.

In the second inhibition experiment, *P*. *destructans* grew optimally in the absence of bacteria, and on media with low initial concentrations of the control bacteria (Fig. 3). By the end of the experiment, the size of *P*. *destructans* colonies differed between bacterial isolates and initial concentrations and the effect of bacterial isolate varied among initial concentrations (S3 and S6 Tables). At the highest initial concentration (10^6^ cells/ml), all bacteria (including the two control bacteria) formed lawns and all reduced growth of *P*. *destructans*. As the starting concentration of the bacteria lawn decreased, fewer isolates significantly reduced the growth of *P*. *destructans*. At the three highest initial bacterial concentrations (10^6^–10^4^ cfu/ml), only isolates PF1, PF2, and PF5 completely suppressed *P*. *destructans* growth for the duration of the experiment (day 42; S6 Table). At the three lowest initial concentrations of the bacteria, where there were relatively few colonies, two *Pseudomonas* isolates, PF1 and PF2 performed significantly better than other isolates in reducing *P*. *destructans* growth and prevented P. *destructans* from growing for the full duration of the experiment (Fig. 3, S6 Table). In both experiments, isolates PF1 and PF2 produced the maximum reduction of mycelial growth across all concentrations, regardless of the way the isolates and *P*. *destructans* were co-cultured.

Cell-free supernatant drawn from liquid bacterial cultures had no effects on the growth of *P*. *destructans*. *Pseudogymnoascus destructans* grew uniformly across all plates, regardless of whether the supernatant added to the plates was from bacteria co-cultured with *P*. *destructans* or cultured alone.

### Bacteria motility and chemotaxis

All seven *Pseudomonas* isolates exhibited signs of motility but no chemotaxis towards *P*. *destructans* colonies was observed. Using microscopy, two of the *Pseudomonas* isolates, PF1 and PF2, were observed dispersing along *P*. *destructans* hyphae. The two control bacteria showed no signs of motility (Table 1).

## Discussion

As the threat of emerging infectious disease grows with increased global travel and trade [5], new ways of managing wildlife disease must be considered [8]. Traditionally, fungal pathogens have been managed using chemical fungicides [39], but toxicity effects on non-target organisms, and application challenges makes it difficult for broad-scale use on wildlife fungal pathogens [29]. The results from our two sets of experiments demonstrate that *in vivo*, bacteria cultured from bats can inhibit the growth of *P*. *destructans*. Our results suggest that augmentation prior to *P*. *destructans* exposure might reduce colonization, whereas bacterial augmentation after exposure could displace *P*. *destructans*. Our results also suggest that a key challenge for successful treatment is applying bacteria such that they will persist on bat skin at high enough concentrations to limit *P*. *destructans* growth below levels that cause lethal disease.

The bacteria we isolated from bats, *Pseudomonas spp*., is ubiquitous in the environment and is well known to have anti-fungal properties [40]. The group of bacteria that these isolates were most closely related to, *Pseudomonas fluorescens*, has previously been detected on several mammals (including bats), as well as amphibians, fish, and plants [41–44]. Members of the *P*. *fluorescens* group are known to produce a suite of antifungal compounds that can inhibit many plant fungal pathogens [45] as well as the amphibian fungal pathogens, *Batrachochytrium dendrobatidis* [46]. Some strains in the *P*. *fluorescens* group are also capable of producing mycolysing enzymes that can colonize the mycelia and conidia of fungi rendering them no longer viable [47]. All of our *Pseudomonas spp*. isolates were motile, which might allow them to use the mycelial networks of fungal colonies to aid in dispersal and colonization [48]. All of these attributes make *P*. *fluorescens* ideal as a proposed candidate to be tested as a biological control agent for reducing infection intensity and increasing survival of bats exposed to *P*. *destructans*.

Whether these antifungal bacteria that naturally occur on bat skin could partially explain differences in mortality from WNS among populations and species is currently unknown. The isolates with strongest inhibitory properties were cultured from *E*. *fuscus*, which has lower mortality from WNS compared to other species [2]. However, we also isolated two strains of *P*. *fluorescens* (PF3 and PF4) that showed moderate *P*. *destructans* inhibition from *M*. *lucifugus*, a species that has suffered severe mortality from WNS[2,20]. Further research is needed to determine the relative abundance, distribution, and inhibitory ability of *P*. *fluorescens* on wild bats and whether presence and abundance of *P*. *fluorescens* influences disease severity.

The next steps in developing a probiotic for WNS should include testing, *in vivo*, one or more of the *P*. *fluorescens* strains that we isolated against *P*. *destructans* using a bat species that suffers high disease mortality from WNS, such as *M*. *lucifugus*, *M*. *septentrionalis*, or *Perimyotis subflavus* [2]. Studies with live hibernating bats will determine whether interactions observed *in vitro* have functional significance in disease outcomes for bat species currently threatened by WNS.

## Supporting Information

S1 Fig Bacterial colony size during first inhibition assay. Colony size of nine bacterial isolates grown on plates inoculated with four different concentrations of *Pseudogymnoascus destructans* with fungal concentrations decreasing from left to right. CHR and SPH are isolates in the genus *Chryseobacterium* and *Sphingomonas* that are not known to produce antifungal compounds. The Control is a sham inoculation of 30% glycerol stock. PF1-7 and PA6 are bacterial isolates in the genus *Pseudomonas*.
(TIF)

S1 Table BLAST results of 16S rRNA sequence from the National Center of Biological Information database for the six bacterial isolated in this study from bats (*Myotis lucifugus* and *Eptesicus fuscus*). (DOCX)

S2 Table AIC values for the first inhibition assay measuring the zone of inhibition produced by bacteria on a lawn of *P*. *destructans*. (DOCX)

S3 Table AIC values for the second inhibition assay measuring how different bacterial isolates influenced the diameter of a *P*. *destructans* colony. (DOCX)

S4 Table AIC values for the first inhibition assay measuring the diameter of bacteria colonies on different concentrations of *P*. *destructans*. (DOCX)

S5 Table Coefficients for linear models of the influence of nine bacterial treatments and a control on the radius of the zones of inhibition of *P*. *destructans* produced by bacteria at four different initial concentrations of P. destructans on day 37 for the data shown in Fig. 2. (DOCX)

S6 Table Coefficients for linear models of the influence of nine bacterial isolates on the diameter of *P*. *destructans* colonies for each bacterial concentration on day 43 for the data shown in Fig. 3. (DOCX)
