# Supplementary figures and images for: Bacteria Isolated from Bats Inhibit the Growth of Pseudogymnoascus destructans, the Causative Agent of White-Nose Syndrome
Source: PLoS One. 2015 Apr 8;10(4):e0121329. doi: 10.1371/journal.pone.0121329 (PMC4390377; doi:10.1371/journal.pone.0121329)

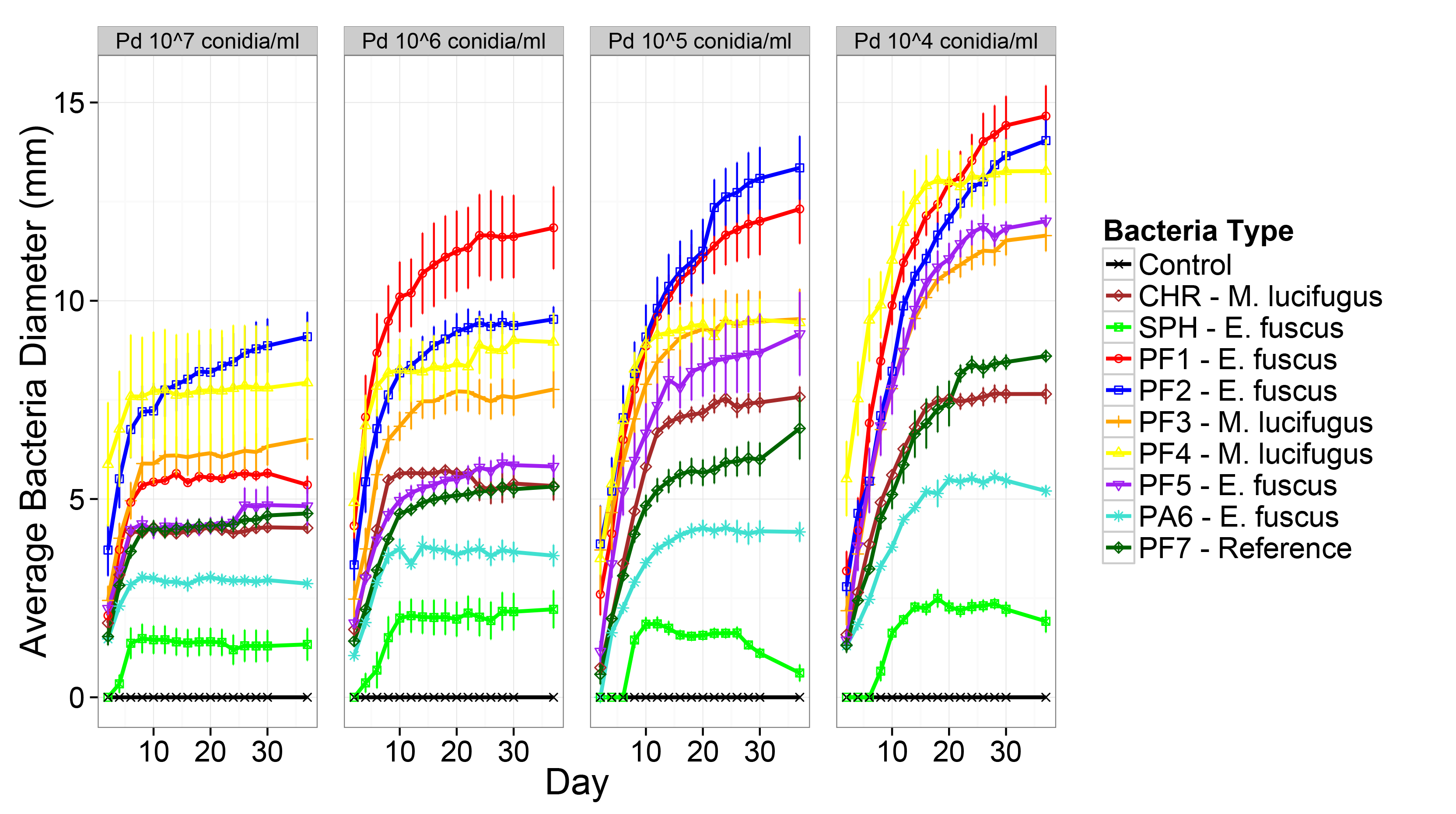

Supplement: S1 Fig — Colony size of nine bacterial isolates grown on plates inoculated with four different concentrations of Pseudogymnoascus destructans with fungal concentrations decreasing from left to right. CHR and SPH are isolates in the genus Chryseobacterium and Sphingomonas that are not known to produce antifungal compounds. The Control is a sham inoculation of 30% glycerol stock. PF1-7 and PA6 are bacterial isolates in the genus Pseudomonas. (TIF) [file pone.0121329.s001.tif]
