# Supplementary material for: Bacteria Isolated from Bats Inhibit the Growth of Pseudogymnoascus destructans, the Causative Agent of White-Nose Syndrome
Source: PLoS One. 2015 Apr 8;10(4):e0121329. doi: 10.1371/journal.pone.0121329 (PMC4390377; doi:10.1371/journal.pone.0121329)
Supplement: S1 Table — (DOCX) [file pone.0121329.s002.docx]

**Table S1.  BLAST results of 16S rRNA sequence from the National Center of Biological Information database with six bacterial isolates from bats (*Myotis lucifugus* and *Eptesicus fuscus*).**

| Bacterial Isolate | Bases | Query cover(%) | Max Id (%) | GenBank accession number match | Bat Species Isolated From |
| --- | --- | --- | --- | --- | --- |
| CHR1 | 1497 | 97 | 98 | KJ123789 | *M. lucifugus* |
| SPH2 | 1464 | 98 | 98 | AY253921.1 | *E. fuscus* |
| PF1 | 1457 | 100 | 100 | D86357 | *E. fuscus* |
| PF2 | 1493 | 100 | 100 | AY850196 | *E. fuscus* |
| PF3 | 1490 | 89 | 99 | AB680974.1 | *M. lucifugus* |
| PF4 | 1497 | 97 | 98 | AB334768.1 | *M. lucifugus* |
| PF5 | 1471 | 100 | 100 | CP000075.1 | *E. fuscus* |
| PA6 | 1453 | 98 | 98 | CP000075.1 | *E. fuscus* |

All e-values were 0.0 (1x10^-127^)
