# Supplementary material for: Bacteria Isolated from Bats Inhibit the Growth of Pseudogymnoascus destructans, the Causative Agent of White-Nose Syndrome
Source: PLoS One. 2015 Apr 8;10(4):e0121329. doi: 10.1371/journal.pone.0121329 (PMC4390377; doi:10.1371/journal.pone.0121329)
Supplement: S2 Table — (DOCX) [file pone.0121329.s003.docx]

**Table S2.  AIC values for the first inhibition assay measuring the zone of inhibition produced by bacteria in a lawn of *P. destructans.***

| Model | AIC | ΔAIC | Weights |
| --- | --- | --- | --- |
| Bacteria type*serial dilution | 27376.89 | 0 | 1.00 |
| Bacteria type +serial dilution | 27602.51 | 225.62 | 0.00 |
| Bacteria type | 27631.6 | 254.71 | 0.00 |
| Serial dilution | 31480.66 | 4103.77 | 0.00 |
| Null | 31486.78 | 4109.89 | 0.00 |
