# Supplementary material for: Bacteria Isolated from Bats Inhibit the Growth of Pseudogymnoascus destructans, the Causative Agent of White-Nose Syndrome
Source: PLoS One. 2015 Apr 8;10(4):e0121329. doi: 10.1371/journal.pone.0121329 (PMC4390377; doi:10.1371/journal.pone.0121329)
Supplement: S4 Table — (DOCX) [file pone.0121329.s005.docx]

**Table S4. AIC values for the second inhibition assay measuring how different bacterial isolates influenced the diameter of a *P. destructans* colony.**

| Model | AIC | ΔAIC | Weights |
| --- | --- | --- | --- |
| Bacteria type*serial dilution | 8440.89 | 0.00 | 1.00 |
| Bacteria type +serial dilution | 8639.34 | 198.45 | 0.00 |
| Bacteria type | 9130.04 | 689.15 | 0.00 |
| Serial dilution | 9702.75 | 1261.86 | 0.00 |
| Null | 10053.62 | 1612.73 | 0.00 |
