# Supplementary material for: Bacteria Isolated from Bats Inhibit the Growth of Pseudogymnoascus destructans, the Causative Agent of White-Nose Syndrome
Source: PLoS One. 2015 Apr 8;10(4):e0121329. doi: 10.1371/journal.pone.0121329 (PMC4390377; doi:10.1371/journal.pone.0121329)
Supplement: S5 Table — (DOCX) [file pone.0121329.s006.docx]

**Table S5: Coefficients for linear models of the influence of nine bacterial treatments and a control on the radius of the zones of inhibition of *P. destructans* produced by bacteria at four different initial concentrations of P. destructans on day 37 for the data shown in Figure 2.**

| *Pseudogymnoascus destructans* concentration 10^4 cfu/ml | | | | |
| --- | --- | --- | --- | --- |
|  | Estimate | Std. Error | t-Value | Pr(>\|t\|) |
| Control | 0.00 | 0.45 | 0.00 | 1.00 |
| CHR | 0.00 | 0.75 | 0.00 | 1.00 |
| SPH | 0.00 | 0.71 | 0.00 | 1.00 |
| PF1 | 9.11 | 0.65 | 13.99 | 0.00 |
| PF2 | 11.72 | 1.05 | 11.20 | 0.00 |
| PF3 | 7.35 | 0.63 | 11.65 | 0.00 |
| PF4 | 7.43 | 0.63 | 11.77 | 0.00 |
| PF5 | 2.19 | 0.63 | 3.48 | 0.00 |
| PA6 | 0.00 | 0.63 | 0.00 | 1.00 |
| PF7 | 4.62 | 0.71 | 6.54 | 0.00 |
|  |  |  |  |  |
| *Pseudogymnoascus destructans* concentration 10^5 cfu/ml | | | | |
|  | Estimate | Std. Error | t value | Pr(>\|t\|) |
| Control | 0.00 | 1.09 | 0.00 | 1.00 |
| CHR | 0.00 | 3.45 | 0.00 | 1.00 |
| SPH | 0.00 | 1.54 | 0.00 | 1.00 |
| PF1 | 17.78 | 1.82 | 9.74 | 0.00 |
| PF2 | 15.88 | 1.72 | 9.21 | 0.00 |
| PF3 | 5.98 | 1.54 | 3.88 | 0.00 |
| PF4 | 12.80 | 1.54 | 8.30 | 0.00 |
| PF5 | 2.70 | 1.65 | 1.64 | 0.11 |
| PA6 | 0.96 | 1.54 | 0.63 | 0.53 |
| PF7 | 4.71 | 1.54 | 3.05 | 0.00 |
|  |  |  |  |  |
| **Pseudogymnoascus destructans* concentration 10^6 cfu/ml | | | | |
|  | Estimate | Std. Error | t value | Pr(>\|t\|) |
| Control | 0.00 | 0.89 | 0.00 | 1.00 |
| CHR | 0.00 | 1.26 | 0.00 | 1.00 |
| SPH | 0.00 | 1.30 | 0.00 | 1.00 |
| PF1 | 23.62 | 1.41 | 16.72 | 0.00 |
| PF2 | 16.17 | 1.35 | 11.98 | 0.00 |
| PF3 | 4.89 | 1.30 | 3.75 | 0.00 |
| PF4 | 9.74 | 1.26 | 7.71 | 0.00 |
| PF5 | 0.99 | 1.30 | 0.76 | 0.45 |
| PA6 | 5.47 | 1.41 | 3.87 | 0.00 |
| PF7 | 0.57 | 1.30 | 0.43 | 0.67 |
|  |  |  |  |  |
| **Pseudogymnoascus destructans* concentration 10^7 cfu/ml | | | | |
|  | Estimate | Std. Error | t value | Pr(>\|t\|) |
| Control | 0.00 | 0.68 | 0.00 | 1.00 |
| CHR | 0.00 | 1.35 | 0.00 | 1.00 |
| SPH | 0.00 | 0.96 | 0.00 | 1.00 |
| PF1 | 32.58 | 1.35 | 24.08 | 0.00 |
| PF2 | 19.29 | 1.07 | 18.04 | 0.00 |
| PF3 | 6.85 | 1.02 | 6.69 | 0.00 |
| PF4 | 13.13 | 0.99 | 13.31 | 0.00 |
| PF5 | 1.16 | 0.96 | 1.21 | 0.23 |
| PA6 | 0.21 | 0.96 | 0.22 | 0.83 |
| PF7 | 0.43 | 0.96 | 0.45 | 0.65 |
