# Supplementary material for: Bacteria Isolated from Bats Inhibit the Growth of Pseudogymnoascus destructans, the Causative Agent of White-Nose Syndrome
Source: PLoS One. 2015 Apr 8;10(4):e0121329. doi: 10.1371/journal.pone.0121329 (PMC4390377; doi:10.1371/journal.pone.0121329)
Supplement: S6 Table — (DOCX) [file pone.0121329.s007.docx]

**Table S6: Coefficients for linear models of the influence of nine bacterial isolates on the diameter of *P. destructans* colonies for each bacterial concentration on day 43 for the data shown in Figure 3.**

| Bacterial concentration 10^1 cfu/ml | | | | |
| --- | --- | --- | --- | --- |
|  | Estimate | Std. Error | t value | Pr(>\|t\|) |
| Control | 14.548 | 0.9159 | 15.88 | 0.00 |
| CHR | -0.1213 | 1.4957 | -0.08 | 0.94 |
| SPH | -2.388 | 1.4957 | -1.60 | 0.13 |
| PF1 | -7.6247 | 1.4957 | -5.10 | 0.00 |
| PF2 | -9.778 | 1.4957 | -6.54 | 0.00 |
| PF3 | -2.5747 | 1.4957 | -1.72 | 0.10 |
| PF4 | -5.1947 | 1.4957 | -3.47 | 0.00 |
| PF5 | -4.3547 | 1.4957 | -2.91 | 0.01 |
| PA6 | -2.2513 | 1.4957 | -1.51 | 0.15 |
| PF7 | 1.237 | 1.7136 | 0.72 | 0.48 |
|  |  |  |  |  |
| Bacterial concentration 10^2 cfu/ml | | | | |
|  | Estimate | Std. Error | t value | Pr(>\|t\|) |
| Control | 14.548 | 1.297 | 11.22 | 0.00 |
| CHR | -0.698 | 2.118 | -0.33 | 0.75 |
| SPH | -7.625 | 2.118 | -3.60 | 0.00 |
| PF1 | -13.338 | 2.118 | -6.30 | 0.00 |
| PF2 | -11.958 | 2.118 | -5.65 | 0.00 |
| PF3 | -7.541 | 2.118 | -3.56 | 0.00 |
| PF4 | -5.088 | 2.118 | -2.40 | 0.03 |
| PF5 | -2.508 | 2.118 | -1.18 | 0.25 |
| PA6 | -4.108 | 2.426 | -1.69 | 0.11 |
| PF7 | -4.818 | 2.118 | -2.28 | 0.03 |
|  |  |  |  |  |
| *Bacterial concentration 10^3 cfu/ml | | | | |
|  | Estimate | Std. Error | t value | Pr(>\|t\|) |
| Control | 14.548 | 0.8576 | 16.96 | 0.00 |
| CHR | -6.432 | 1.2128 | -5.30 | 0.00 |
| SPH | -7.724 | 1.2128 | -6.37 | 0.00 |
| PF1 | -13.866 | 1.2128 | -11.43 | 0.00 |
| PF2 | -13.514 | 1.2128 | -11.14 | 0.00 |
| PF3 | -13.042 | 1.2128 | -10.75 | 0.00 |
| PF4 | -12.474 | 1.2128 | -10.29 | 0.00 |
| PF5 | -12.258 | 1.2128 | -10.11 | 0.00 |
| PA6 | -3.706 | 1.2128 | -3.06 | 0.00 |
| PF7 | -9.342 | 1.2128 | -7.70 | 0.00 |
|  |  |  |  |  |
| *Bacterial concentration 10^4 cfu/ml | | | | |
|  | Estimate | Std. Error | t value | Pr(>\|t\|) |
| Control | 14.548 | 0.3537 | 41.13 | 0.00 |
| CHR | -10.81 | 0.5002 | -21.61 | 0.00 |
| SPH | -8.868 | 0.5002 | -17.73 | 0.00 |
| PF1 | -14.548 | 0.5002 | -29.08 | 0.00 |
| PF2 | -14.548 | 0.5002 | -29.08 | 0.00 |
| PF3 | -14.278 | 0.5002 | -28.54 | 0.00 |
| PF4 | -11.344 | 0.5002 | -22.68 | 0.00 |
| PF5 | -14.548 | 0.5002 | -29.08 | 0.00 |
| PA6 | -3.9 | 0.5002 | -7.80 | 0.00 |
| PF7 | -12.376 | 0.5002 | -24.74 | 0.00 |
|  |  |  |  |  |
| *Bacterial concentration 10^5 cfu/ml | | | | |
|  | Estimate | Std. Error | t value | Pr(>\|t\|) |
| Control | 14.548 | 0.3703 | 39.29 | 0.00 |
| CHR | -14.023 | 0.5554 | -25.25 | 0.00 |
| SPH | -9.732 | 0.5237 | -18.58 | 0.00 |
| PF1 | -14.548 | 0.5237 | -27.78 | 0.00 |
| PF2 | -14.548 | 0.5237 | -27.78 | 0.00 |
| PF3 | -14.548 | 0.5554 | -26.19 | 0.00 |
| PF4 | -14.266 | 0.5237 | -27.24 | 0.00 |
| PF5 | -14.548 | 0.5237 | -27.78 | 0.00 |
| PA6 | -5.936 | 0.5237 | -11.34 | 0.00 |
| PF7 | -13.958 | 0.5237 | -26.65 | 0.00 |
|  |  |  |  |  |
| *Bacterial concentration 10^6 cfu/ml | | | | |
|  | Estimate | Std. Error | t value | Pr(>\|t\|) |
| Control | 14.548 | 0.4461 | 32.61 | 0.00 |
| CHR | -14.548 | 0.7285 | -19.97 | 0.00 |
| SPH | -7.998 | 0.7285 | -10.98 | 0.00 |
| PF1 | -14.548 | 0.7285 | -19.97 | 0.00 |
| PF2 | -14.548 | 0.7285 | -19.97 | 0.00 |
| PF3 | -14.548 | 0.7285 | -19.97 | 0.00 |
| PF4 | -12.733 | 0.8346 | -15.26 | 0.00 |
| PF5 | -14.548 | 0.7285 | -19.97 | 0.00 |
| PA6 | -5.5347 | 0.7285 | -7.60 | 0.00 |
| PF7 | -14.548 | 0.7285 | -19.97 | 0.00 |
